# Refractive and visual function changes in twilight conditions

**DOI:** 10.1371/journal.pone.0267149

**Published:** 2022-04-15

**Authors:** Tatsuya Iizuka, Takushi Kawamorita, Tomoya Handa, Hitoshi Ishikawa

**Affiliations:** 1 Kitasato University Graduate School of Medical Sciences, Sagamihara, Kanagawa, Japan; 2 Department of Orthoptics and Visual Science, Kitasato University School of Allied Health Sciences, Sagamihara, Kanagawa, Japan; 3 Department of Ophthalmology, Kitasato University School of Medicine, Sagamihara, Kanagawa, Japan; Keio University School of Medicine, JAPAN

## Abstract

**Purpose:**

We investigated the effect of luminance on refraction and visual function under twilight conditions.

**Methods:**

Twenty young adults (mean age 20.5 ± 0.5 years) without ocular diseases and 20 eyes were included in the study. Subjective and objective spherical equivalent power (SE), logMAR, pupil diameter, ocular aberration, and ocular axial length were evaluated. Measurements were conducted in a light room with high luminance (300 cd/m^2^) targets (photopic), in a dark room with low luminance (10 cd/m^2^) targets (twilight), and a dark room after 15 min of adaptation to low luminance (10 cd/m^2^) targets (after adaptation: AA). Differences between the three conditions were analyzed using the Friedman test and Scheffe’s multiple comparisons.

**Results:**

The results of logMAR were -0.20 ± 0.07, -0.08 ± 0.08, and -0.11 ± 0.08 in photopic, twilight, and AA, respectively, with significant differences between photopic and twilight (p < 0.001) and between photopic and AA (p < 0.001). Then subjective SE were -3.58 ± 2.04 D, -3.75 ± 2.08 D, and -3.74 ± 2.04 D in photopic, twilight, and AA, respectively, with significant differences between photopic and twilight (p = 0.007) and photopic and AA (p = 0.023). However, none of the other objective SEs produced a significant difference (p = 0.63). The pupil diameter and ocular aberration changed significantly in all conditions (p < 0.001).

**Conclusions:**

Subjective myopic refraction increased and visual resolution decreased in younger subjects. However, this change in refraction is less than one level (±0.25 D) in clinical optometry, so fully corrected eyeglasses are important when assuming refraction in twilight, and there is no need for additional correction.

## Introduction

According to a report by the World Health Organization, approximately 1.35 million people worldwide die due to road traffic accidents [1]. Traffic fatalities are increasing worldwide; however, they are decreasing annually in Japan. The highest number of traffic accidents in Japan was reported by the Japanese National Police Agency in 2004 when 952,720 accidents occurred and 1,183,617 people were injured. Since then, the number of traffic accidents and injuries has been decreasing every year, and in 2020, there were 309,178 traffic accidents and 369,476 injuries. However, these are not small numbers and continue to remain high (S1 Fig) [2]. According to a report by the National Police Agency of Japan, the main causes of traffic accidents in Japan are misuse of steering wheels and brakes, inattention to the road ahead, and errors in judgment due to dangerous objects or lack of safety [3]. These factors are influenced by the time required to see dangerous objects and pedestrians, suggesting a relationship with visual function [4]. Furthermore, when examining the time of occurrence of traffic accidents, it has been reported that traffic accidents with casualties occur more frequently during the sunset hours of 16:00–18:00, a time when large fluctuations in luminance occur due to sunset (S2 Fig) [5].

Studies investigating traffic safety in the twilight at sunset have reported mental fatigue, stress, and inability to cope with sudden changes in luminance to affect driver reaction time. These are therefore considered risk factors for drivers’ safety worldwide [6]. As a way to adjust to the reduction of luminance during sunset, pupils dilate and this process can cause increased ocular aberrations and significantly reduced retinal image quality [7]. In general, high-order aberrations within ocular aberrations cannot be corrected by eyeglasses or contact lenses, and these phenomena are considered to be one of the issues of quality of vision as they adversely affect the ocular optical system [8].

Additionally, functional changes occur over time at the retinal cell level, from photopic vision, in which cone cell function is dominant, to scotopic vision, in which rod cell function is dominant, to mesopic vision, in which both cone and rod cells function during adaptation [9, 10] These eye functional changes suggest that twilight is an underlying problem associated with traffic accidents at sunset.

Mesopic vision is a switch between photopic and scotopic vision, and visual acuity is maintained by the interaction of cone and rod cells (see [11]). However, there are no standardized conditions for evaluating mesopic vision. Most studies on twilight vision show luminance of 1 cd/m^2^ or less, and studies assuming slightly brighter twilight vision show luminance of 3–5 cd/m^2^ or less. When sunset is assumed to be in twilight, these studies have a luminance that is closer to nighttime [12–15]. We measured the luminance of white lines, road signs, and road surfaces on roads finclusioor 30 min before and after sunset to conduct our research assuming everyday scenes. The analysis images and results are shown in S3 and S4 Figs. The luminance of the road immediately after sunset was 10cd/m^2^ which dropped further to 1cd/m^2^, thirty minutes after sunset. To study the effects of refraction and visual function in young people under low-luminance twilight conditions, in this study, experiments were conducted under low-luminance (10 cd/m^2^) twilight conditions reproduced in the laboratory.

## Methods

### Participants

Twenty young adults with corrected visual acuity > 20/20 without ocular diseases other than refractive error were included in the study. Their mean age (± standard deviation: SD) was 20.5 ± 0.5 years. Participants were selected based on the absence of history of intraocular surgery or disease affecting the pupil diameter. If astigmatism and myopia were present, individuals with astigmatism correction of -1.75 Diopter (D) or less (for cylindrical lenses) and -8.75 D or less myopia (for spherical lenses) were included in the study. This study was conducted in accordance with the Declaration of Helsinki, with the approval of the Ethics Committee of the School of Allied Health Sciences of Kitasato University (2019–011), and written informed consent was obtained from all participants. Furthermore, consent was obtained from the participants of the study to publish the photographs in the article.

### Experimental setting

**Fig 1:**
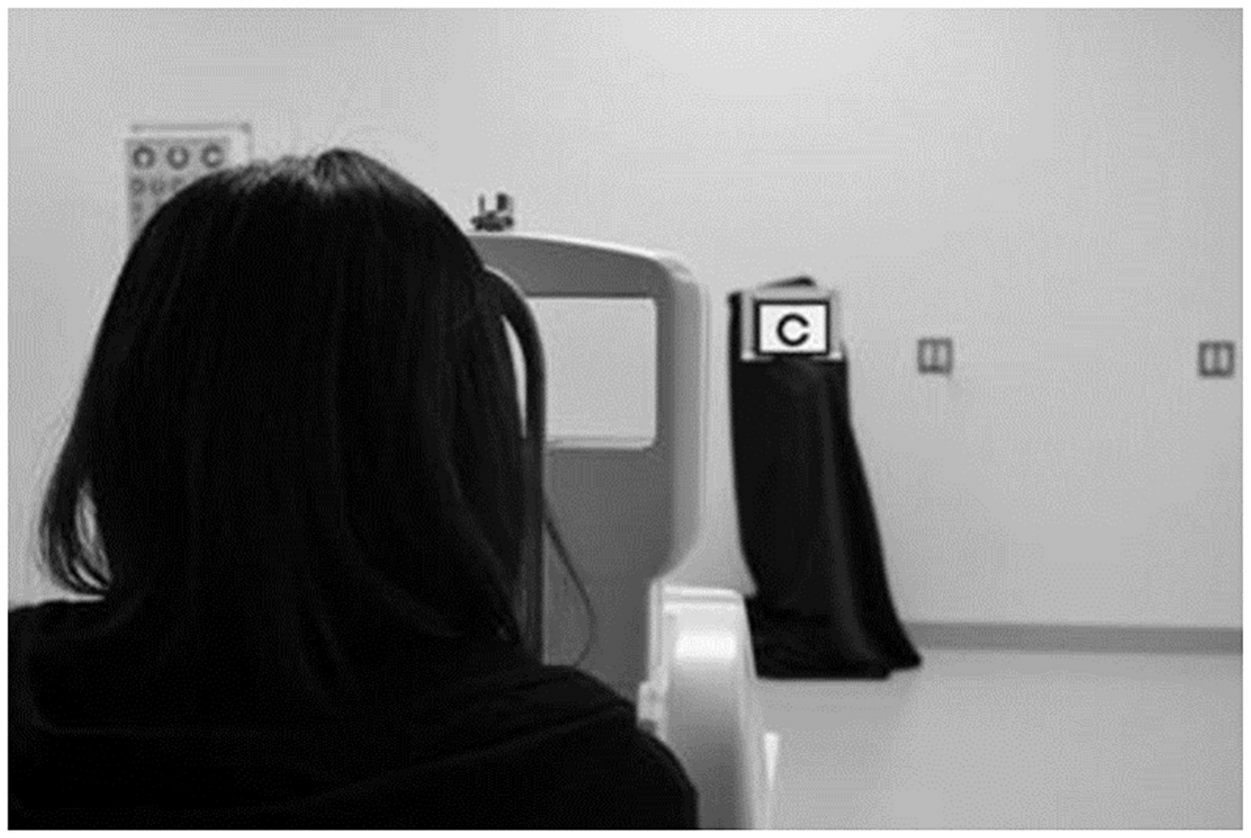
Experimental scene in lightroom (measurement with a binocular open auto refractometer). A luminance-adjustable tablet terminal is set up at a distance of 5 m in front of the eyes of the research participants, presenting the Landolt rings, and measuring the subjective and objective refractive errors.

Two different environments were used to investigate changes in visual function and refraction. In the lightroom, the environment was similar to that of a typical vision test: 300 cd/m^2^ of visual target luminance and 500 lx of horizontal illuminance. In the darkroom, the luminance measurements at sunset shown in the supplementary figure were used as a reference, and the measurements were conducted in an environment with visual target luminance of 10 cd/m^2^, lights turned off to avoid fluctuations in pupil diameter when looking directly at the lights, and horizontal illuminance of 0.01 lx or less. We set up a luminance-adjustable tablet iPad 4th generation (Apple) at a distance of 5 m in front of the participant’s eyes and displayed the Landolt ring targets (Fig 1).

### Measuring equipment and method

Only 1 eye from each participant was examined in this study. For each subject ID, the measurement eye was pre-determined for left and right randomization. Optometry lenses available on the market were used for subjective refraction measurements, and the cross-cylinder method was used for astigmatism correction. Visual acuity and subjective refraction were measured by using a logMAR (Logarithm of the Minimum Angle of Resolution) chart. Five Landolt rings corresponding to the logMAR chart were displayed on an iPad screen. Participants verbally stated the letter they saw on the screen, and responses were recorded by the researcher. The logMAR was lowered incrementally when 5/5 correct Landolt rings were responded to, and visual acuity and subjective and subjective refraction were measured. Entries with less than four correct responses among the five Landolt rings were rejected, and the logMAR with five correct responses before one step was adopted as visual acuity and refraction. For the subjective refractive values, the refractive values of the most plus spherical and cylinder power at the maximum visual acuity when all five answers of the Landolt ring were correct were adopted as the perfect refractive correction.

To measure objective refraction and pupil diameter, the average value of six consecutive measurements was used with a binocular open auto refractometer WAM-5500 (Shigiya Seiki Seisakusho Co., Ltd., Hiroshima, Japan). Subjective and objective refraction measurements were taken under three different conditions: high luminance (300 cd/m^2^) visual targets in a bright room (hereinafter called “photopic”), low luminance (10 cd/m^2^) visual targets in a dark room (hereinafter called “twilight”), and low luminance (10 cd/m^2^) visual targets in a dark room 15 min after adaptation (hereinafter called “after adaptation: AA”). Photopic and twilight measurements were taken in random order, but AA was measured after twilight. The refraction was examined for spherical power, cylinder power, and equivalent spherical power (SE). Subjective refraction measurements were performed by the same optometrist. We used OA-1000 (Tomey) for ocular axial length measurements and OPD-Scan II ARK-10000 (Nidek) for ocular aberration measurements. To calculate the number of high-order aberrations corresponding to the natural pupil diameter, pupil diameters obtained under three different conditions were used as the analyzed diameters, and the Zernike coefficients up to the sixth order term were analyzed. The Schwiegerling Algorithm was used for eyes with pupil diameters greater than 6 mm to recalculate the Zernike coefficients from an analytical diameter of 6 mm to any analytical diameter with a pupil diameter greater than 6 mm [16]. For ocular aberrations, we examined low-order (Defocus only), total high-order, coma (S3+S5), and spherical aberrations (S4+S6).

### Statistical analysis

BellCurve for Excel (Social Survey Research Information Co., Ltd., Tokyo, Japan) was used for the statistical analysis. Visual acuity and refractive changes in photopic, twilight, and AA were analyzed using the Friedman test, and Scheffe’s multiple comparisons was performed to analyze the differences between conditions. To examine the factors affecting the change in visual acuity and refraction in photopic and twilight conditions, we conducted a stepwise forward selection procedure using multiple regression analysis. The change in subjective SE was the dependent variable; the independent variables were defocus, total higher-order aberration, coma aberration, spherical aberration, pupil diameter, and ocular axial length. When the change in logMAR was the dependent variable, the independent variables were defocus, total higher order aberration, coma aberration, and spherical aberration. All variables, except the ocular axial length, represent the amount of change from photopic to twilight. Both models, visual acuity and refraction, were run backward-forward, stepwise, with all variables first put into the model. At each step, a variable was excluded from the model if it did not have a statistically significant contribution (p > 0.10). Finally, the remaining variables were identified as strong factors. Results were considered statistically significant at P < 0.05.

## Results

**Fig 2:**
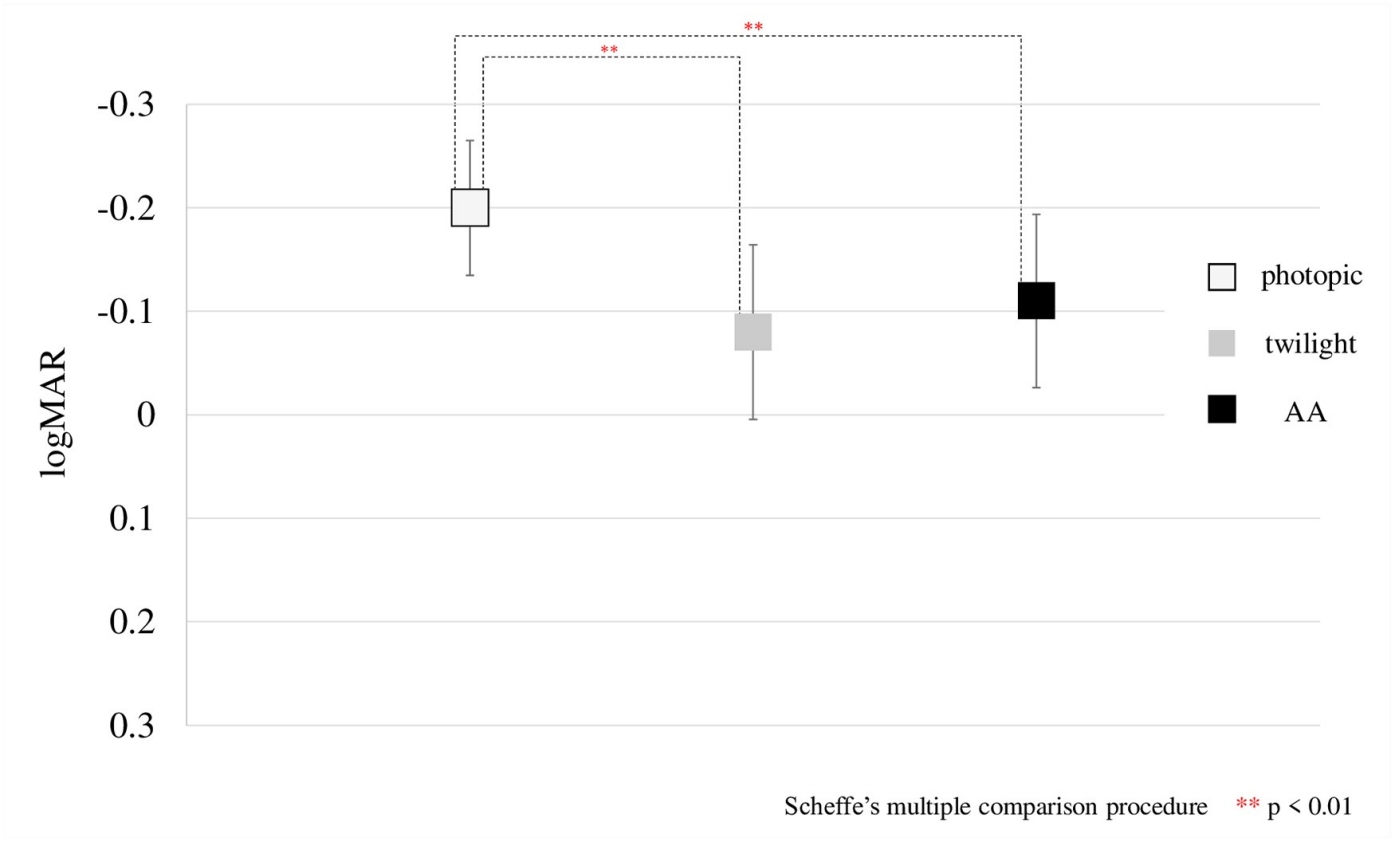
Results of logMAR visual acuity in photopic and twilight and AA are shown. When the results of these three conditions are analyzed using the Friedman test, p<0.001 is obtained. Therefore, Scheffe’s multiple comparison procedures are performed, and the difference is significant at p<0.001 between photopic and twilight, and p<0.001 between photopic and AA.

**Fig 3:**
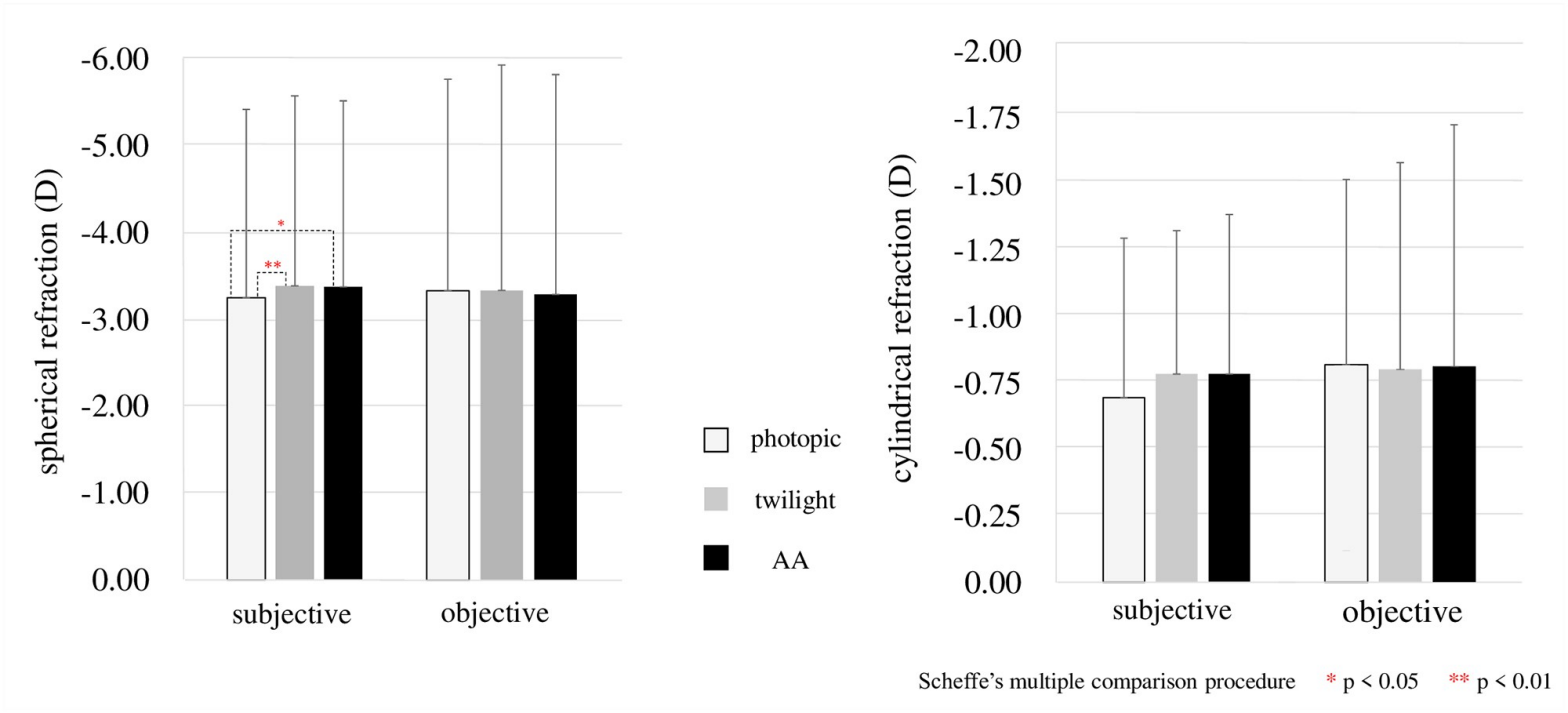
Results of subjective and objective spherical and cylindrical refraction in photopic and twilight and AA are shown. When the results of these three conditions are analyzed using the Friedman test, only subjective spherical refraction shows a difference between the conditions, p = 0.005. Therefore, when Scheffe’s multiple comparison procedure is performed, there is a significant difference in subjective spherical refraction, p = 0.009, between photopic and twilight conditions, and p = 0.049 between photopic and AA.

**Fig 4:**
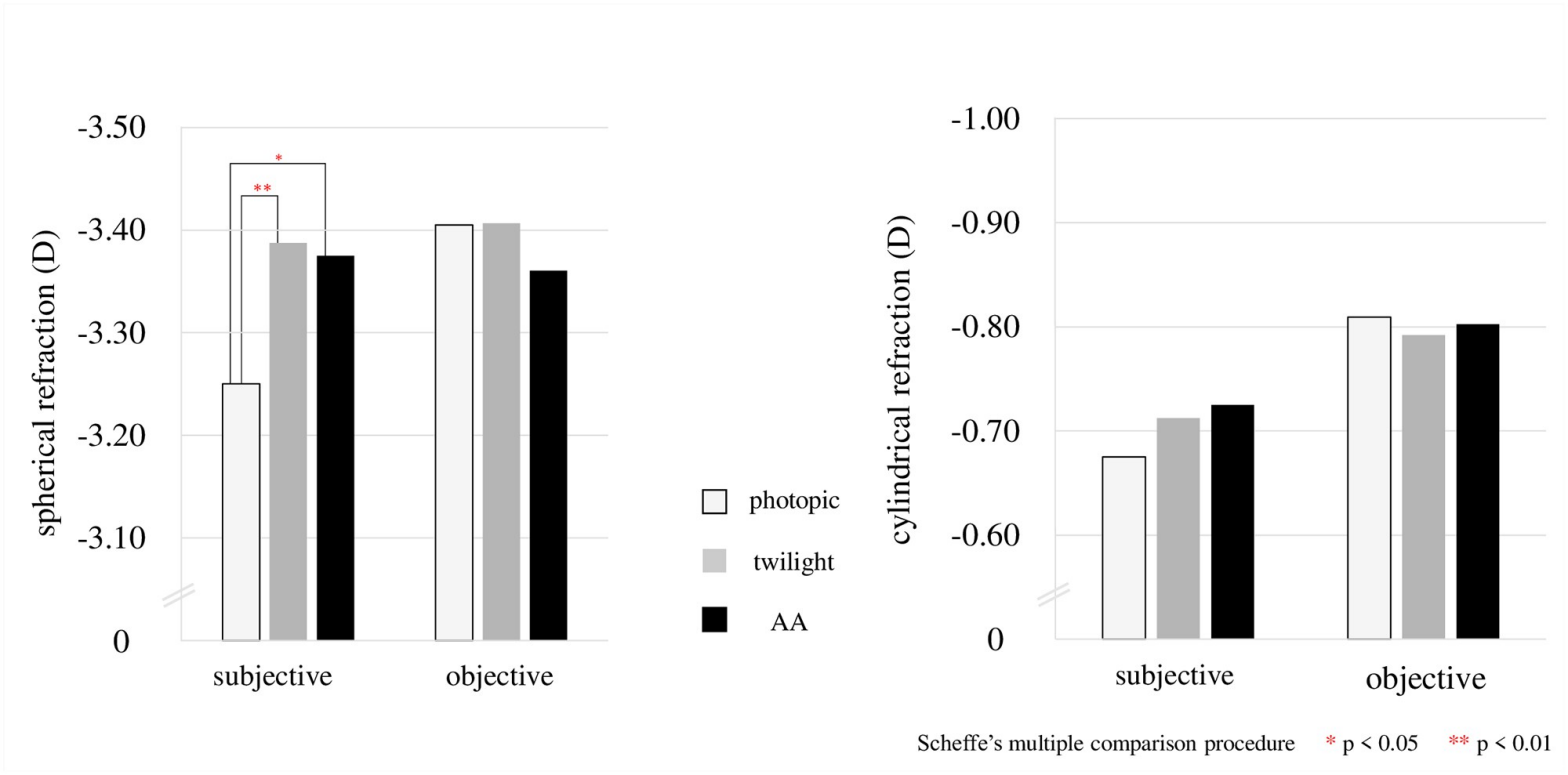
Enlarged comparison of the results shown in Fig 3.

**Fig 5:**
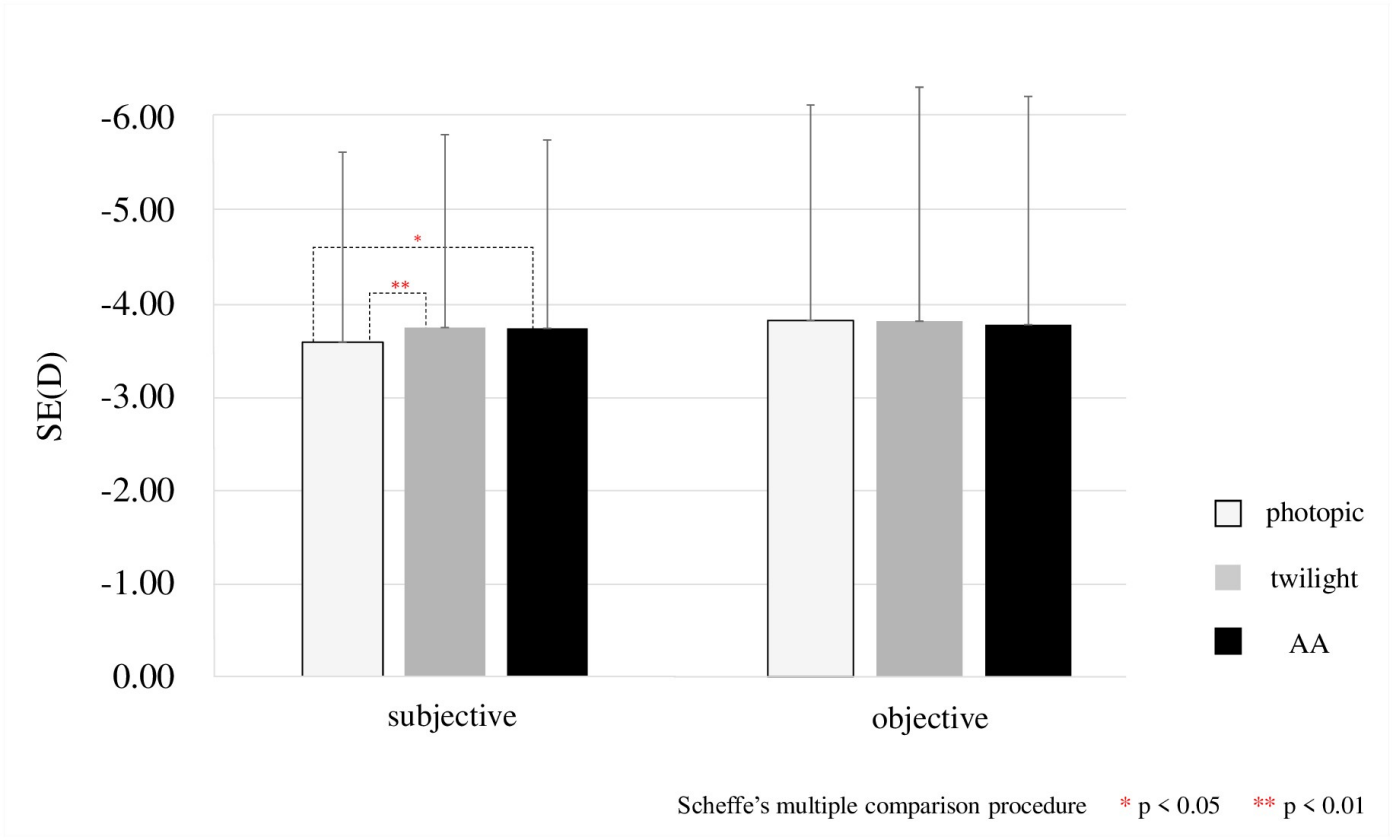
Results of subjective and objective SE in photopic and twilight and AA are shown. When the results of these three conditions are analyzed using the Friedman test, only subjective SE shows a difference between the conditions, p = 0.003. Therefore, when Scheffe’s multiple comparison procedure is performed, there is a significant difference in subjective SE, p = 0.007, between photopic and twilight conditions, and p = 0.023 between photopic and AA.

**Fig 6:**
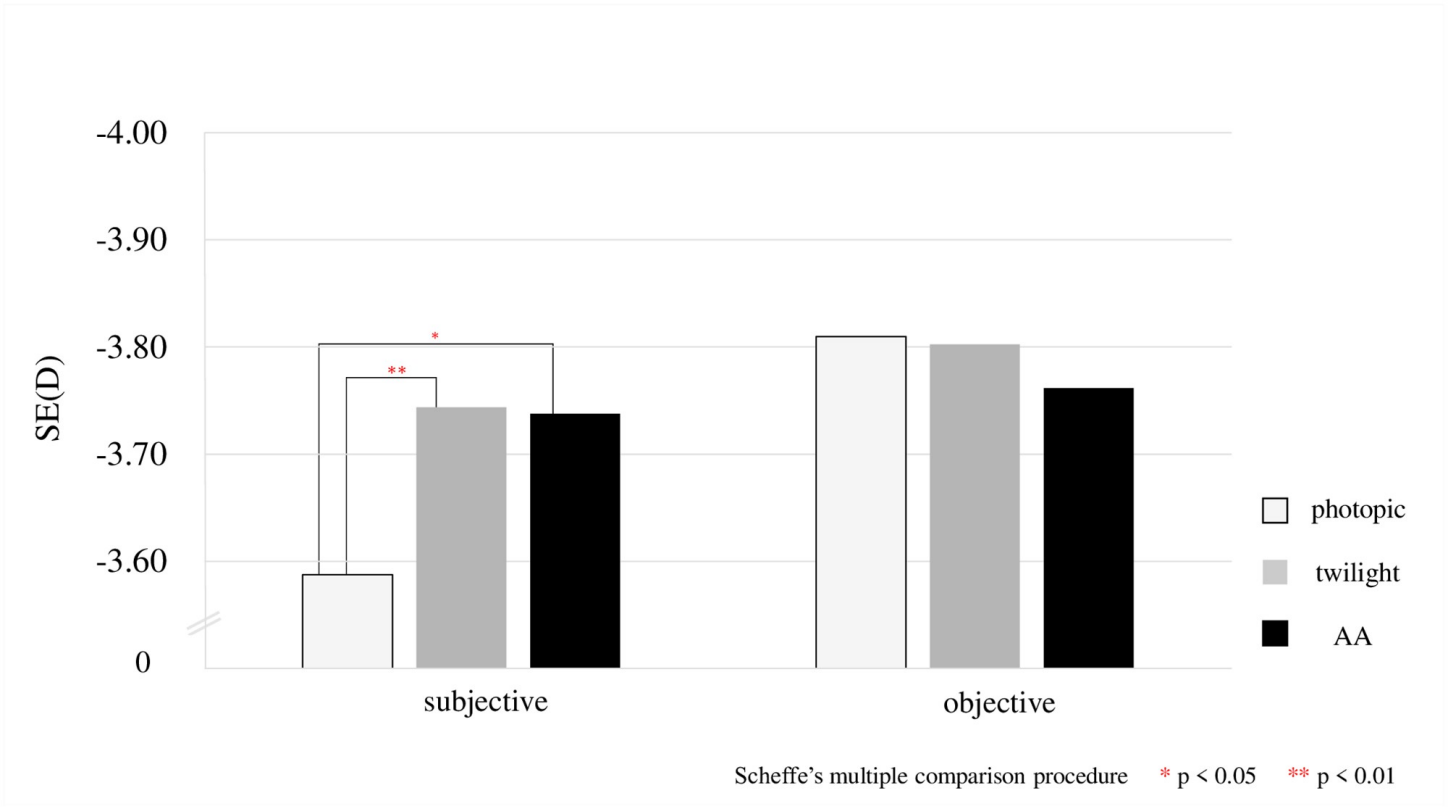
Enlarged comparison of the results shown in Fig 5.

The results for photopic, twilight, and AA are shown in the Table 1 below. The logMAR (visual acuity) was -0.20 ± 0.07 in photopic, -0.08 ± 0.08 in twilight, and -0.11 ± 0.08 AA, with significant differences between photopic and twilight (p < 0.001) (Scheffe’s multiple comparison procedure) and between photopic and AA (p < 0.001) (Fig 2). For subjective refraction, spherical power was -3.25 ± 2.16 D in photopic, -3.38 ± 2.16 D in twilight, and -3.36 ± 2.12 D AA, with significant differences between photopic and twilight (p = 0.009) and photopic and AA (p = 0.049) (Figs 3 and 4). Subjective SE was -3.58 ± 2.04 D in photopic, -3.75 ± 2.08 D in twilight, and -3.74 ± 2.04 D AA, with significant differences between photopic and twilight (p = 0.007) and photopic and AA (p = 0.023) (Figs 5 and 6). Individual subjective SE and logMAR results are presented in S1 Table for each of the three conditions. There were significant differences in pupil diameter, defocus, higher-order aberration, spherical aberration, and coma aberration in all conditions. (p < 0.001). There were no significant differences in subjective cylinder power (p = 0.06), objective spherical power (p = 0.34), objective cylinder power (p = 0.39), or objective SE (p = 0.63) in any condition (Friedman test). The ocular axial length was 24.50±1.11 mm.

**Table 1:** Measurement data of the study participants (n = 20).

				(mean±SD)
Parameter	p	photopic	twilight	AA
logMAR	**< 0.001	-0.20±0.07	-0.08±0.08	-0.11±0.08
subjective spherical refraction (D)	** 0.005	-3.25±2.16	-3.38±2.16	-3.36±2.12
objective spherical refraction (D)	0.34	-3.40±2.48	-3.40±2.65	-3.36±2.58
subjective cylindrical refraction(D)	0.06	-0.68±0.61	-0.75±0.66	-0.76±0.69
objective cylindrical refraction(D)	0.39	-0.80±0.69	-0.79±0.77	-0.80±0.92
subjective SE (D)	** 0.003	-3.58±2.04	-3.75±2.08	-3.74±2.04
oubjective SE (D)	0.63	-3.81±2.32	-3.80±2.50	-3.76±2.44
pupil diameter (mm)	**< 0.001	4.71±0.89	6.64±0.72	6.03±0.94
defocus(μm)	**< 0.001	3.04±2.10	6.02±3.87	5.15±3.89
total high order aberration (μm)	**< 0.001	0.44±0.43	1.02±0.79	0.75±0.65
coma aberration (μm)	**< 0.001	0.39±0.34	0.84±0.62	0.64±0.53
spherical aberration (μm)	**< 0.001	0.20±0.27	0.53±0.54	0.36±0.41

SE indicates spherical equivalent power; AA, after adaptation; logMAR, logarithm of the minimum angle of resolution. Friedman test.

** p < 0.01.

The stepwise multivariate regression analysis of refraction showed that the pupil diameter (standardized partial regression coefficient β = -0.745, p < 0.001) was significantly associated with change in subjective SE (adjusted R^2^ = 0.532, VIF = 1.00). Variables excluded from the multivariate regression model were ocular axial length (p = 0.999), defocus (p = 0.932), total high order aberration (p = 0.343), coma aberration (p = 0.531), and spherical aberration (p = 0.193).

Stepwise multivariate regression analysis of visual acuity showed that the total high-order aberration (standardized partial regression coefficient β = 0.395, p = 0.027) was significantly associated with change in logMAR (adjusted R^2^ = 0.127, VIF = 1.00). Variables excluded from the multivariate regression model included, coma aberration (p = 0.657), and spherical aberration (p = 0.984).

## Discussion

In twilight and AA, the subjective refractive power was significantly shifted myopic. Ocular aberrations increased significantly with pupil diameter proliferation; however, the increase in aberrations did not affect refraction. Since there was no significant difference in objective refractive power, the involvement of accommodation by crystalline lens changes was negative. There was no night myopia caused by refractive changes due to accommodation. These results indicate that the myopic defocus caused by subjective refractive power was perceived as a blur as the depth of focus became shallower with pupil dilation. It was suggested that the refractive power fluctuated depending on the amount of blur awareness [17].

Night myopia is a typical phenomenon of refractive changes caused by changes in luminance. Since the 1900s, this phenomenon has been variously discussed as being caused by spherical aberration, chromatic aberration, and accommodation by the crystalline lens [18–20]. Recent studies have shown that spherical and chromatic aberrations are unlikely to affect nighttime myopia, and the most likely explanation is the accommodation errors that occur in very low luminance conditions [21, 22]. In our results in twilight and AA, the change in spherical aberration was not related to refraction. In the twilight environment assumed in this study, the luminance was 10 cd/m^2^, which is slightly bright for night myopia to occur, suggesting that no significant change in objective refractive power occurred [23].

Low-order aberrations were significantly increased in twilight and AA; however, there was no relationship with subjective refractive power. It has been reported that the acceptable range of blur due to low-order aberration differs from person to person depending on age, visual impairment, and individual personality, and it has been suggested that refractive changes for subjective blur vary among individuals even in twilight [24–26].

If we assume that these are real-life situations, the pupils dilate with the rapid decrease in illuminance after sunset, and we become aware of a slight blur. In traffic-related studies on refraction, blur has a detrimental effect on driving as drivers are delayed in recognizing road signs and pedestrians when +0.50 D is loaded [27, 28]. In this study, the subjective refractive error that changed from photopic to twilight was -0.17 D on average, which is smaller than the ±0.25 D that is one step of power correction in general optometry. Therefore, although a statistically significant difference was detected, this result was not considered a meaningful change when applied to clinical ophthalmology. However, if optical correction with eyeglasses or contact lenses is not appropriate, it is anticipated that the refraction would increase further in the twilight, which would delay the visualization of dangerous objects and pedestrians. In summary, wearing inappropriate eyeglasses is unsuitable for driving at twilight. Particularly eyeglasses need to be fully corrected in photopic vision, and there is no need for additional refractive correction.

In terms of logMAR visual acuity, there was a one-step decrease from bright to twilight, similar to the logMAR visual acuity measured at a visual target luminance of 3 cd/m^2^ in a similar twilight study [13]. A decrease in logMAR visual acuity of two or three levels has been reported in mesopic vision with a visual target luminance of less than 1 cd/m^2^ [12]. Therefore, when establishing a method for evaluating visual function in the twilight as an assumption for driving at sunset, luminance between 3 cd/m^2^ and 10 cd/m^2^ is considered to be similar to that of actual twilight. Under the experimental conditions used, changes in visual acuity at twilight were not significantly influenced by refraction or higher-order aberrations. Instead we believe they are due to luminance- and color temperature-dependent cone cell dysfunction, which cannot be corrected (other than by controlling the luminance of the object being viewed). Owing to these effects, vision is dependent on luminance [29]. Therefore, in the hours after twilight, road signs and signs warning of dangerous objects on the road should have a certain level of luminance, which will improve visibility while driving and prevent accidents.

One limitation of this study is its inability to reproduce actual changes in sunset luminance over time. However, if we can examine the effects of the darkest criteria at sunset, we can envision the changes that may have the most adverse effects. In addition, since subjective visual acuity and refraction at 10 cd/m^, which was set as twilight, required 3–5 minutes of measurement time, this created a slight acclimation and did not accommodate the instantaneous luminance changes.

## Supporting information

S1 Fig Number of traffic accidents and injuries from 1948 to 2020 according to statistics from the National Police Agency in Japan, which peaked in 2004 and has continued to decline annually since. (PDF)

S2 Fig Number of elementary and junior high school students injured in traffic accidents in 2019 by Japan’s National Police Agency. Traffic accidents are most common between 4:00 p.m. and 6:00 p.m.
(PDF)

S3 Fig (a) Location for analyzing luminance before and after sunset (Kitasato, Minami-ku, Sagamihara-shi, Kanagawa, Japan: 35-32-14N, 139-23-43E, measured on December 4, 2020 at 16:00–17:00). (b) Luminance measurement and analysis are performed at the location shown in (a) using a two-dimensional luminance measurement analysis ACE3-1000 (HI-LAND Co., Ltd., Tokyo, Japan). The measurement points are the road sign (arrow 1 in the figure), the road surface (arrow 2), the white line (1) (arrow 3), the yellow line (arrow 4), and the white line (2) (arrow 5).
(PDF)

S4 Fig Analysis results of the luminance of each measurement point. At sunset, the luminance of the road surface and road signs is 10 cd/m2, while 15 and 30 min after sunset, all the luminance are less than 10 cd/m2, and less than 1 cd/m2.
(PDF)

S1 Table Subjective refraction and visual acuity measurement data for each subject (n = 20). (PDF)

## References

[1] World Health Organization. Global status report on road safety. 2018. https://www.who.int/violence_injury_prevention/road_safety_status/2018/en/. Accessed July 7 2021.

[2] National Police Agency. The white paper on police. https://www.npa.go.jp/hakusyo/r03/index.html. Accessed July 7 2021; 2019.

[3] National Police Agency. Fatal accident. https://www.e-stat.go.jp/stat-search/file-download?statInfId=000032051740&fileKind=2. Accessed July 7 2021; 2020.

[4] Enke K. Possibilities for improving safety within the driver vehicle environment loop, 7th international Technical Conference on Experimental Safety Vehicle, Paris; 1979.

[5] *Institute for Traffic Accident Research and Data Analysis*. *Traffic Statistics*. https://www.itarda.or.jp/materials/traffic/free. Accessed July 7 2021; 2019 Edition.

[6] González-Hernández B, Usami DS, Prasolenko O, Burko D, Galkin A, Lobashov O, et al. The driver’s visual perception research to analyze pedestrian safety at twilight. *Transportation Research Procedia*. 2020; 45:827–834. doi: 10.1016/j.trpro.2020.02.087

[7] De Groot SG, Gebhard JW. Pupil size as determined by adapting luminance. *J Opt Soc Am*. 1952 July;42(7):492–495. doi: 10.1364/josa.42.000492 . 14939111

[8] Marcos S. Aberrometry: basic science and clinical applications. Bull Soc Belge Ophtalmol. 2006;(302):197–213. . 17265799

[9] Frumkes TE, Sekuler MD, Reiss EH. Rod-cone interaction in human scotopic vision. *Science*. 1972 February 25;175(4024):913–914. doi: 10.1126/science.175.4024.913 5008610

[10] Zele AJ, Maynard ML, Feigl B. Rod and cone pathway signaling and interaction under mesopic illumination. *J Vis*. 2013 January 16;13(1):21. doi: 10.1167/13.1.21 . 23325348

[11] Buck SL. Rod-cone interactions in human vision. In: Chalupa LM, Werner JS, eds. *The Visual Neurosciences*; vol 1. Cambridge MA: MIT Press; 2004:863–878.

[12] Heinrich SP, Blechenberg T, Reichel C, Bach M. The “speed” of acuity in scotopic vs. photopic vision. *Graefes Arch Clin Exp Ophthalmol*. 2020 December;258(12):2791–2798. doi: 10.1007/s00417-020-04867-6 Epub August 15 2020. 32803325 PMC7677280

[13] Lin RJ, Ng JS, Nguyen AL. Determinants and standardization of mesopic visual acuity. *Optom Vis Sci*. 2015 May;92(5):559–565. doi: 10.1097/OPX.0000000000000584 . 25906409

[14] Puell MC, Palomo C, Sánchez-Ramos C, Villena C. Normal values for photopic and mesopic letter contrast sensitivity. *J Refract Surg*. 2004 September-October;20(5):484–488. doi: 10.3928/1081-597X-20040901-12 . 15523961

[15] Pesudovs K, Marsack JD, Donnelly WJ, III, Thibos LN, Applegate RA. Measuring visual acuity—mesopic or photopic conditions, and high or low contrast letters? *J Refract Surg*. 2004 September-October;20(5):S508–S514. . 15523967

[16] Schwiegerling J. Scaling Zernike expansion coefficients to different pupil sizes. *J Opt Soc Am A Opt Image Sci Vis*. 2002 October;19(10):1937–1945. doi: 10.1364/josaa.19.001937 . 12365613

[17] Tucker J, Charman WN. The depth-of-focus of the human eye for Snellen letters. *Am J Optom Physiol Opt*. 1975 January;52(1):3–21. doi: 10.1097/00006324-197501000-00002 . 1111286

[18] Koomen M, Tousey R, Scolnik R. The spherical aberration of the eye. *J Opt Soc Am*. 1949 May;39(5):370–376. doi: 10.1364/josa.39.000370 . 18131435

[19] Otero JM. Influence of the state of accommodation on the visual performance of the human eye. *J Opt Soc Am*. 1951 December;41(12):942–948. doi: 10.1364/josa.41.000942 . 14908733

[20] Leibowitz HW, Owens DA. Night myopia and the intermediate dark focus of accommodation. *J Opt Soc Am*. 1975 October;65(10):1121–1128. doi: 10.1364/josa.65.001121 . 1185296

[21] Artal P, Schwarz C, Cánovas C, Mira-Agudelo A. Night myopia studied with an adaptive optics visual analyzer. *PLOS ONE*. 2012;7(7):e40239. doi: 10.1371/journal.pone.0040239 Epub July 2 2012. 22768343 PMC3388063

[22] Chirre E, Prieto PM, Schwarz C, Artal P. Night myopia is reduced in binocular vision. *J Vis*. 2016 June 1;16(8):16. doi: 10.1167/16.8.16 . 27333457

[23] Charman WN. Night myopia and driving. *Ophthalmic Physiol Opt*. 1996 November;16(6):474–485. . 8944194

[24] Legge GE, Mullen KT, Woo GC, Campbell FW. Tolerance to visual defocus. *J Opt Soc Am A*. 1987 May;4(5):851–863. doi: 10.1364/josaa.4.000851 . 3598739

[25] Kline DW, Buck K, Sell Y, Bolan TL, Dewar RE. Older observers’ tolerance of optical blur: age differences in the identification of defocused text signs. *Hum Factors*. 1999 September;41(3):356–364. doi: 10.1518/001872099779611049 . 10665204

[26] Woods RL, Colvin CR, Vera-Diaz FA, Peli E. A relationship between tolerance of blur and personality. *Invest Ophthalmol Vis Sci*. 2010 November;51(11):6077–6082. doi: 10.1167/iovs.09-5013 Epub May 26 2010. 20505192 PMC2948623

[27] Wood JM, Collins MJ, Chaparro A et al. Differential effects of refractive blur on day and nighttime driving performance. *Invest Ophthalmol Vis Sci*. 2014 April 9;55(4):2284–2289. doi: 10.1167/iovs.13-13369 . 24618322

[28] Wood JM, Marszalek R, Carberry T, Lacherez P, Collins MJ. Effects of different levels of refractive blur on nighttime pedestrian visibility. *Invest Ophthalmol Vis Sci*. 2015 July;56(8):4480–4485. doi: 10.1167/iovs.14-16096 . 26193924

[29] Wilcox WW. The basis of the dependence of visual acuity on illumination. *Proc Natl Acad Sci U S A*. 1932 January;18(1):47–56. doi: 10.1073/pnas.18.1.47 16577428 PMC1076158

