# Supplementary figures and images for: Refractive and visual function changes in twilight conditions
Source: PLoS One. 2022 Apr 15;17(4):e0267149. doi: 10.1371/journal.pone.0267149 (PMC9012392; doi:10.1371/journal.pone.0267149)

# Supplementary Figure S1.

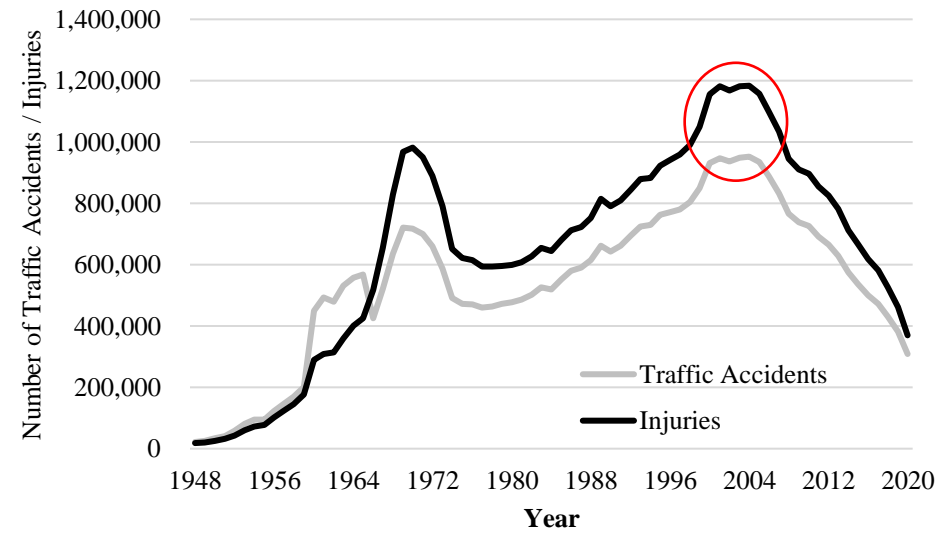

Supplement: S1 Fig — (PDF) [file pone.0267149.s001.pdf]

# Supplementary Figure S2.

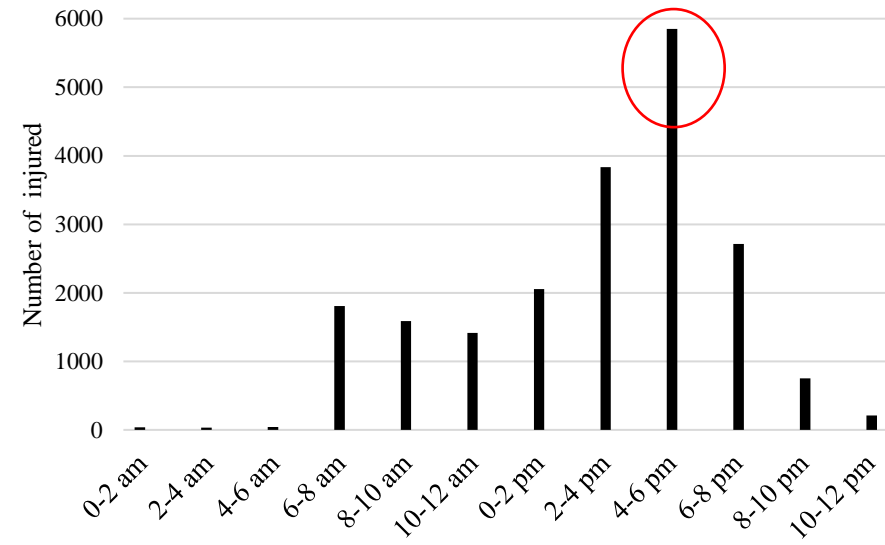

Supplement: S2 Fig — Traffic accidents are most common between 4:00 p.m. and 6:00 p.m. (PDF) [file pone.0267149.s002.pdf]

# Supplementary Figure S3.

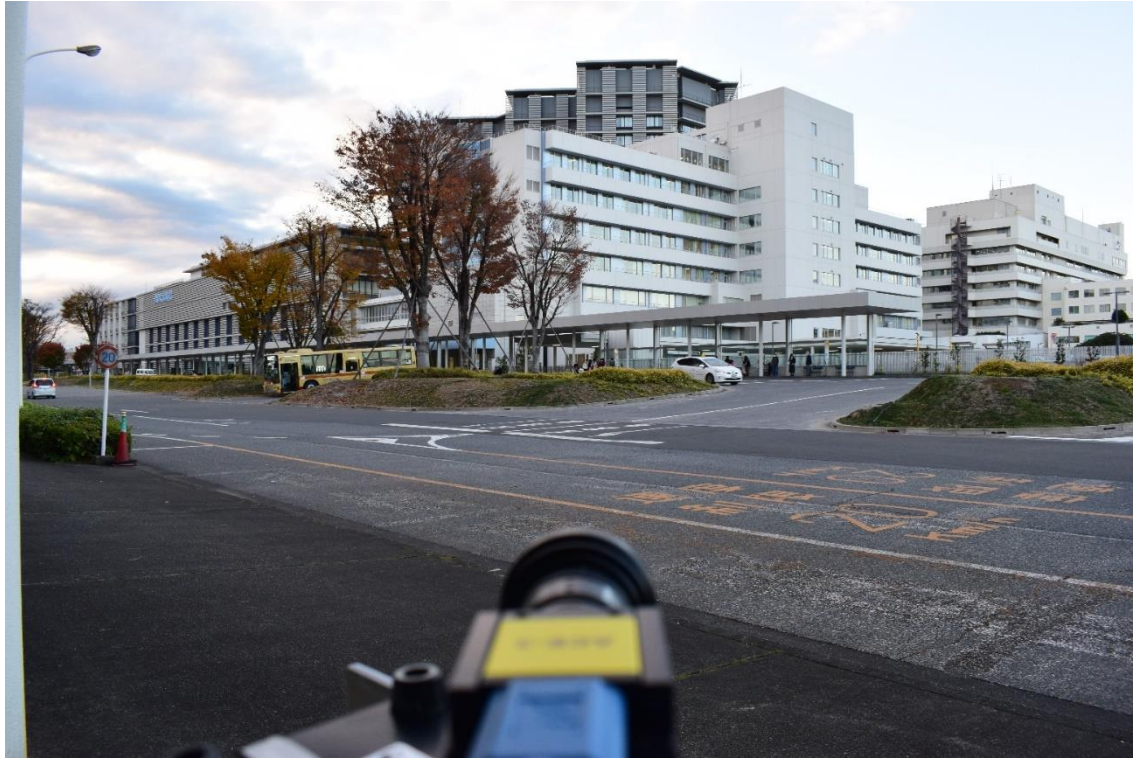

( a )

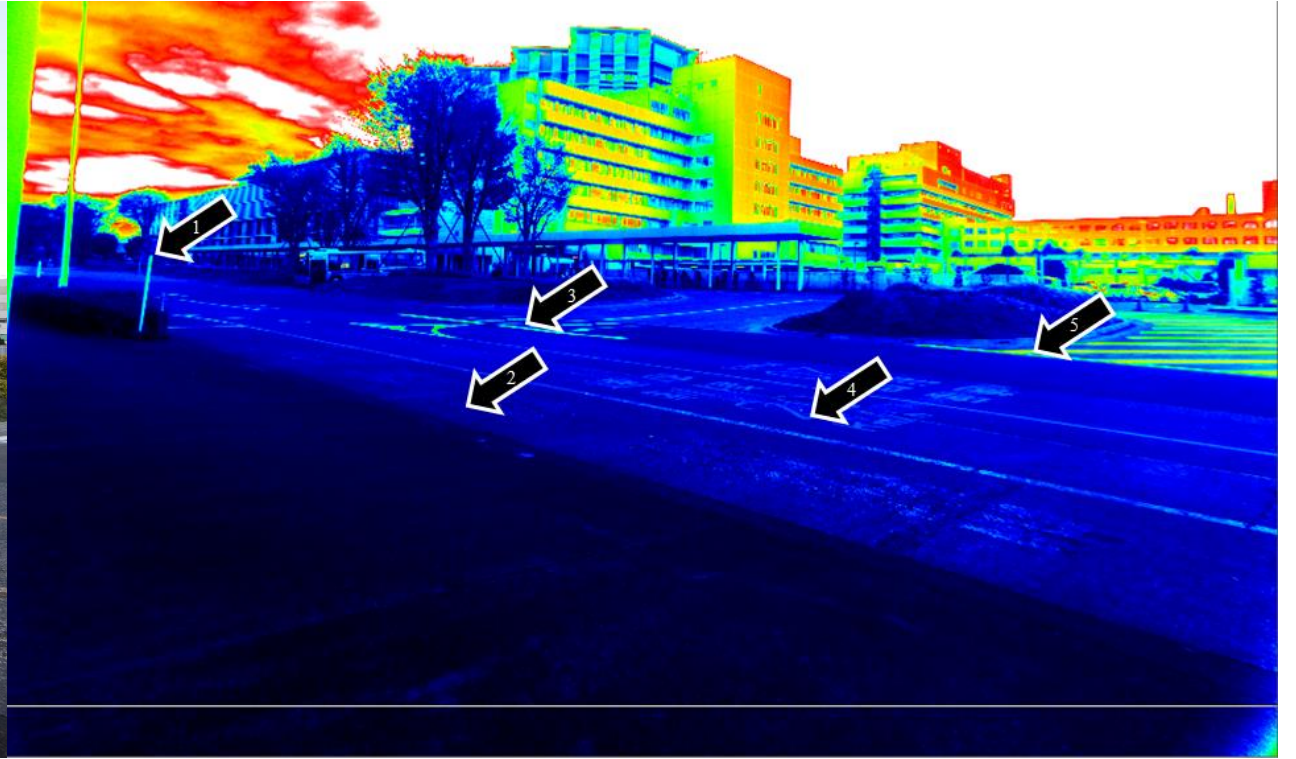

( b )

Supplement: S3 Fig — (a) Location for analyzing luminance before and after sunset (Kitasato, Minami-ku, Sagamihara-shi, Kanagawa, Japan: 35-32-14N, 139-23-43E, measured on December 4, 2020 at 16:00–17:00). (b) Luminance measurement and analysis are performed at the location shown in (a) using a two-dimensional luminance measurement analysis ACE3-1000 (HI-LAND Co., Ltd., Tokyo, Japan). The measurement points are the road sign (arrow 1 in the figure), the road surface (arrow 2), the white line (1) (arrow 3), the yellow line (arrow 4), and the white line (2) (arrow 5). (PDF) [file pone.0267149.s003.pdf]

# Supplementary Figure S4.

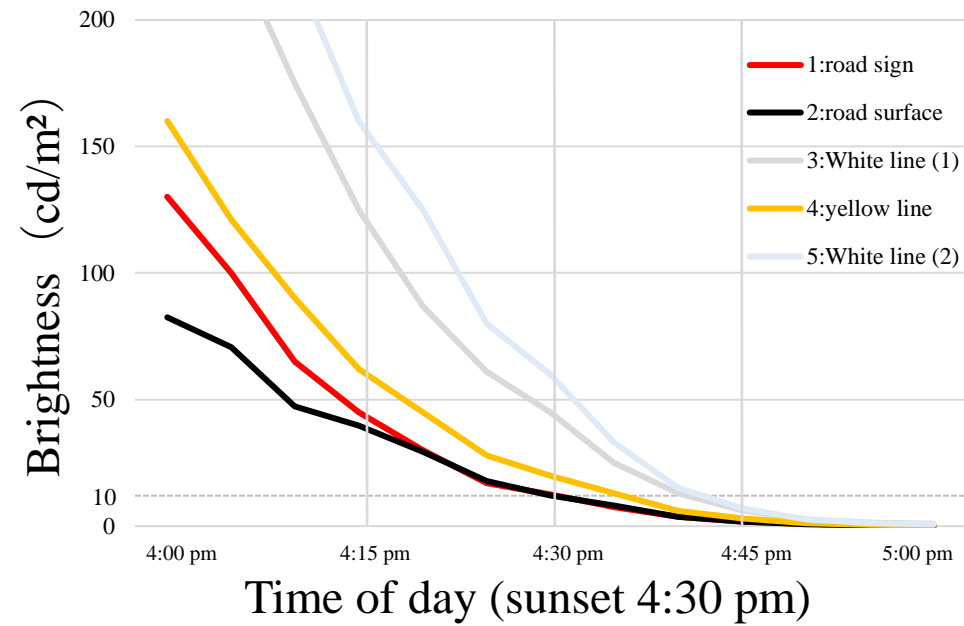

Supplement: S4 Fig — At sunset, the luminance of the road surface and road signs is 10 cd/m2, while 15 and 30 min after sunset, all the luminance are less than 10 cd/m2, and less than 1 cd/m2. (PDF) [file pone.0267149.s004.pdf]
