# Supplementary material for: Refractive and visual function changes in twilight conditions
Source: PLoS One. 2022 Apr 15;17(4):e0267149. doi: 10.1371/journal.pone.0267149 (PMC9012392; doi:10.1371/journal.pone.0267149)
Supplement: S1 Table — (PDF) [file pone.0267149.s005.pdf]

Supplementary Table 1. Subjective refraction and visual acuity measurement data for each subject (n = 20)

	subjective SE (D)			logMAR		
Subjects(eyes)No	photopic	twilight	AA	photopic	twilight	AA
1	-0.75	-1.00	-0.75	-0.20	0.10	-0.10
2	-0.75	-0.75	-0.75	-0.30	-0.10	-0.20
3	-0.88	-1.00	-1.13	-0.10	-0.10	-0.10
4	-0.88	-0.88	-1.13	-0.30	-0.30	-0.30
5	-1.00	-1.00	-1.25	-0.20	0.00	0.00
6	-1.88	-2.13	-2.13	-0.30	-0.10	-0.10
7	-2.63	-2.63	-2.63	-0.20	-0.20	-0.20
8	-2.88	-3.13	-3.13	-0.20	-0.20	-0.20
9	-3.00	-3.25	-3.00	-0.20	0.00	0.00
10	-3.75	-4.00	-4.25	-0.30	0.00	-0.10
11	-4.00	-4.25	-4.50	-0.30	0.00	-0.10
12	-4.13	-4.38	-4.38	-0.10	0.00	0.00
13	-4.38	-4.63	-4.38	-0.20	-0.10	-0.10
14	-4.75	-4.75	-4.75	-0.20	-0.10	-0.20
15	-4.88	-5.25	-5.50	-0.20	-0.10	-0.20
16	-5.25	-5.25	-5.00	-0.10	-0.10	0.00
17	-6.13	-6.13	-5.88	-0.10	-0.10	-0.10
18	-6.25	-6.63	-6.63	-0.20	-0.10	-0.10
19	-6.38	-6.63	-6.38	-0.10	0.00	-0.10
20	-7.25	-7.38	-7.38	-0.20	-0.10	-0.10

SE indicates spherical equivalent power; AA, after adaptation;
logMAR, logarithm of the minimum angle of resolution.
